# Experimental investigation on the hazard of geyser created by an entrapped air release in baffle-drop shafts

**DOI:** 10.1038/s41598-023-34253-1

**Published:** 2023-05-16

**Authors:** Qinghua Yang, Qian Yang

**Affiliations:** 1 School of Civil Engineering, Southwest Jiaotong University, Chengdu, 610031 Sichuan China; 2 China MCC5 Group Corp. Ltd., Chengdu, 610063 Sichuan China

**Keywords:** Civil engineering, Natural hazards

## Abstract

The geyser phenomenon seriously threatens the safe operation of deep tunnel drainage systems and drop shaft structural safety. To simulate the geyser process in a baffle-drop shaft, a 1:50 scale model test system was used to research the response relationship between the geyser mechanism and test parameters such as water depth, inlet pressure, and inlet volume. The results show that the pressure in a baffle-drop shaft fluctuates sharply during the geyser process. This is caused by the release of a high-pressure air mass, and high-speed movement of the air–water mixture causes a local pressure imbalance in the drop shaft. A prediction formula for the maximum geyser height of a baffle-drop shaft was established by a multiple linear regression model. Geyser occurrence conditions for the baffle-drop shaft were proposed combined with the response relationship between different influence variables and geyser intensity. Except for the inlet pressure, submerged state of the baffles, and measured location, the hydrodynamic load on the bottom of the baffles is also related to the randomness of the air–water mixture jetted on the baffle bottom. The maximum hydrodynamic load on the baffle bottom during the geyser is 10 times the hydrodynamic load on the baffle surface under normal discharge conditions. This research provides a theoretical reference for the structural design and safe operation of baffle-drop shafts.

## Introduction

**Figure 1:**
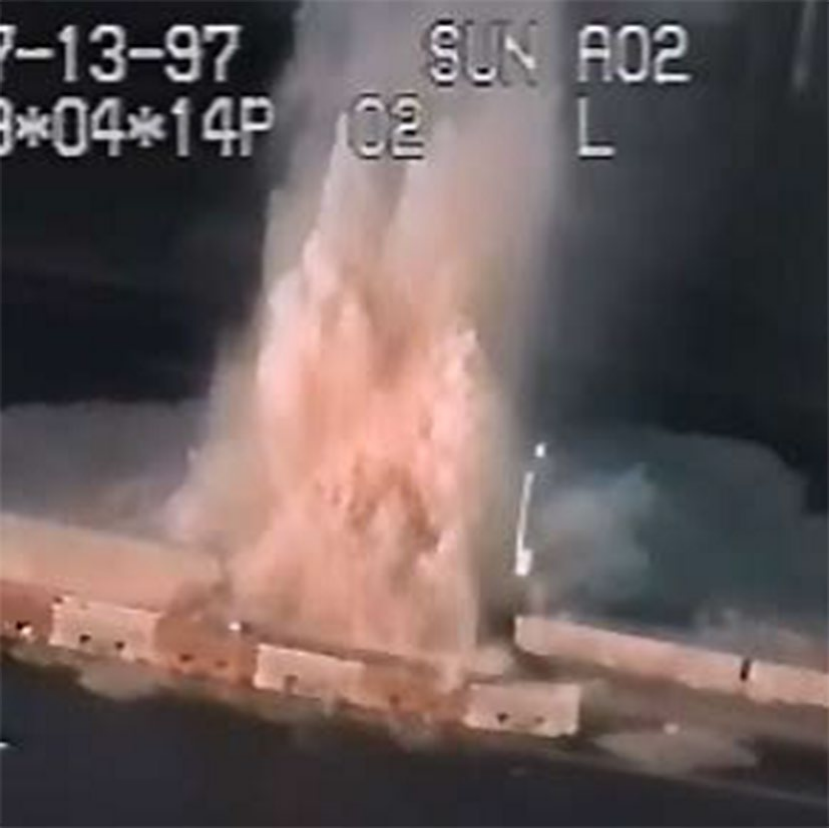
Photograph of geyser events. (Photo by Chris Ellis, University of Minnesota St. Anthony Falls Laboratory).

In recent years, deep tunnel drainage systems have been used to mitigate the impact of intense rain events and reduce the frequency of overflow pollution events in urban areas^1^. However, deep tunnel drainage systems can quickly fill with water flow under intense rainfall conditions, and a large amount of air in the main tunnel can be squeezed into the atmosphere through the drop shaft. However, a considerable amount of air can remain in a deep tunnel system because some air is not pushed out in time, and additional air can be carried into the system with the water flow. This air quickly forms a high-pressure entrapped air mass under the action of transient flow and can move back and forth in the main tunnel with pressure differences. When the entrapped air moves to an outlet pipe connecting the drop shaft and main tunnel, this high-pressure air mass can be suddenly released due to buoyancy, resulting in a large amount of air–water mixture being sprayed into the atmosphere to form a geyser^2,3^. As shown in Fig. 1, this violent release of entrapped air can initiate rainwater and sewage overflows, which can cause traffic delays and affect pedestrian safety. In addition, the strong impact load of the air–water mixture can lead to the destruction of tunnel and shaft structures, which can seriously threaten the safe operation of deep tunnel drainage systems^4^.

In the 1980s, surging problems in tunnel and reservoir plan (TARP) deep tunnels in Chicago, USA were investigated by Song et al.^5,6^ and He et al.^7^. Through mathematical model analysis, a series of measures, such as limiting the inflow, adding a reservoir at the downstream end, adjusting the initial water volume and installing a surge shaft at the upstream end, were proposed to solve these surging problems. In addition, Guo et al.^8^ noted that most geyser events were caused by the inertial impact force of rising water. Therefore, only hydrodynamics were adopted in their research, and the influence of air–water interactions on surges and geysers was ignored. However, increasing evidence has shown that entrapped air releases in deep tunnel drainage systems under heavy rainfall are the main cause of geysers and structural damage. The violent geyser mechanism of a vertical shaft completely full of water was experimentally and numerically investigated by Leon^9,10^. This result indicated that the spill of water at the top of the shaft was caused by a rising air pocket, which resulted in a significant pressure gradient between the vertical shaft and the horizontal pipe, and the greatest intensity in terms of geyser height occurred at the third, fourth or even the fifth eruption^11,12^. According to laboratory observations, Vasconcelos et al.^13^ noted that the buoyancy of large air pockets caused the water moving upwards in a shaft to form a geyser. Moreover, field observations suggested that high-pressure air may be a significant component of a water jet during a geyser event^2,14^. Using experiments and numerical simulations, Vasconcelos et al.^15^ proposed that air–water interactions may play an important role in a tunnel filling process, and the effect of the air phase on the water motion cannot be neglected in poorly ventilated deep tunnel systems. A multiphase flow numerical model was applied by Shao et al.^16^ to capture both air and water dynamics and simulate a geyser. Zhou et al.^17^ experimentally investigated the transient flow characteristics of horizontal pipes containing trapped air during a rapid filling process. Atrabi et al.^18^ also studied the motion regularities of entrapped air cavities in an inclined pipe. In addition, the hydraulic characteristics of different drop shafts were experimentally investigated by some scholars^19–21^. Many studies have demonstrated that previously entrapped air in water flows in deep tunnel drainage systems plays a crucial role in the formation of a geyser.

In summary, the influence of entrapped air and air–water interactions cannot be ignored in geyser research^22,23^. To a certain extent, the above results have enhanced the understanding of surges and geysers in deep tunnel drainage systems. However, there is no research on the geyser phenomenon of baffle-drop shafts at present. Different from ordinary cylindrical shafts, the baffle-drop shafts are divided into wet and dry sides by a vertical dividing wall. Similar to a cylindrical shaft, the dry side is used for ventilation and equipment channels; however, there are many staggered baffles on the wet side, which can weaken geyser intensity. This indicates that a geyser can be caused by a rising Taylor bubble^24^, but a regular Taylor bubble cannot be formed in the semicircular dry side of a baffle-drop shafts. Therefore, when a geyser occurs, the air–water two-phase flow characteristics in a baffle-drop shaft are actually different from those in an ordinary cylindrical shaft. In addition, the connected area between the dry and wet sides at the bottom of the dividing wall plays a decisive role in the eruption intensity of a geyser. If this area is too large, it can steer the geyser to release on the dry side and affect traffic and pedestrian safety. However, if the area is too small, a large amount of air–water mixture will spray into the wet side and cause structural damage to the baffles at the shaft bottom.

Due to the lack of details about geyser phenomenon into baffle-drop shaft, a 1:50 scale model test system based on Froude similitude was implemented to observe the geyser process, analyse the variation law of jet velocity and pressure at the shaft top, measure the water impact load of baffles at the shaft bottom, and investigate the response relationship between the geyser intensity and influence factors such as air pressure, air volume, pressure gradient, water depth, and connected area. An in-depth examination of high-speed air–water two-phase flow characteristics in baffle-drop shafts during the geyser process can provide a theoretical basis for further understanding geyser mechanisms and the safe design of shaft structures.

## Experimental method

### Experimental facility

**Figure 2:**
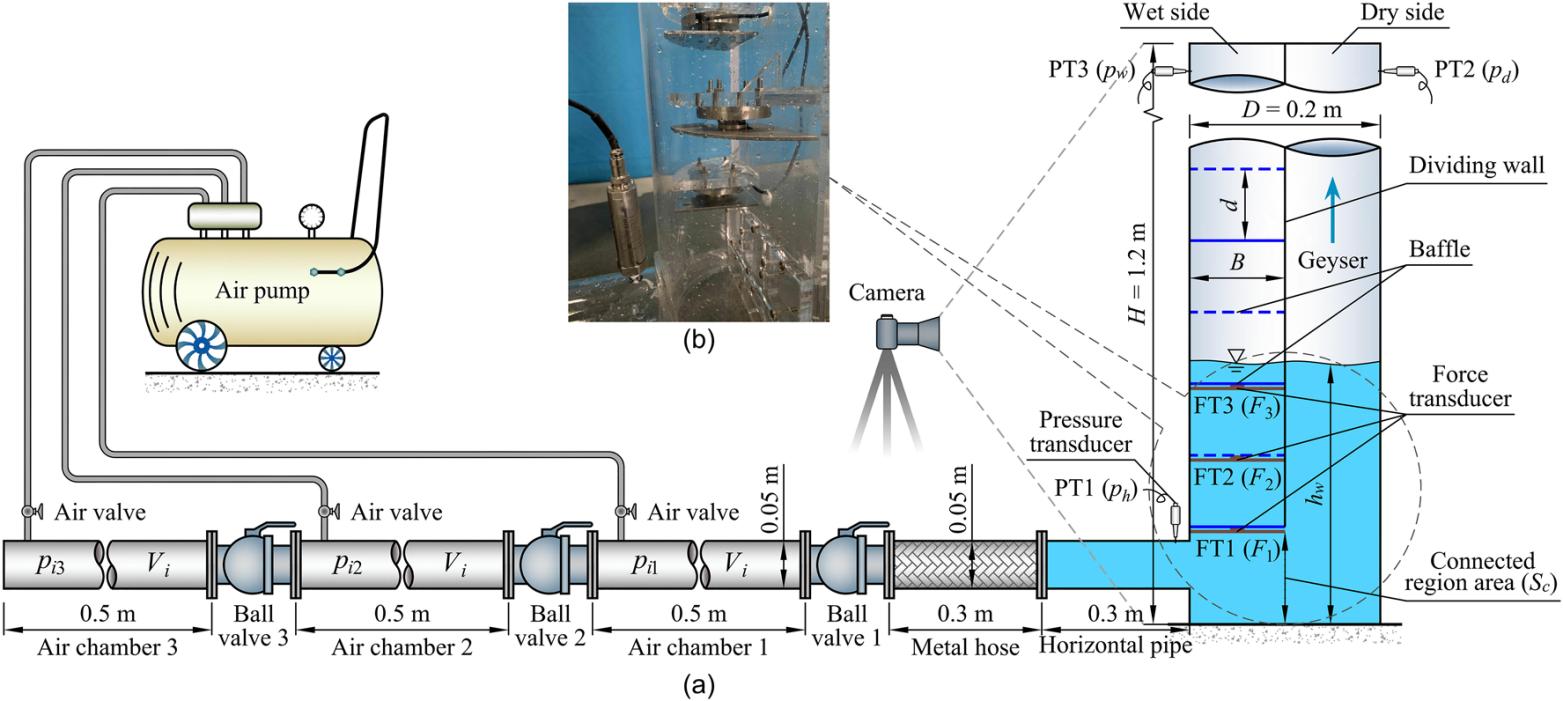
Experimental system for releasing a large amount of entrapped air into a baffle-drop shaft: (**a**) sketch of the experimental setup; (**b**) photo of the shaft bottom. PT, pressure transducer; FT, force transducer.

Wright et al.^25^ noted that several variables, including shaft diameter, height, air pressure, and water depth, can synergistically cause a geyser. To realistically reproduce the geyser process, an experimental system for releasing a large amount of entrapped air into a baffle-drop shaft was established and is shown in Fig. 2. The system consisted of a plexiglass shaft model, horizontal pipe, air chambers, ball valves, and air pump. The physical model was designed with a length scale of 1:50 based on the prototype shaft applied in the Donghaochong deep tunnel drainage system in Guangzhou, China. The shaft height was *H* = 1.2 m, and the shaft diameter was *D* = 2*B* = 0.2 m, where *B* is the baffle width. According to previous results^26^, the optimal size of the baffle-drop shaft was *d*/*B* = 0.485, in which *d* is the baffle spacing. The baffles were installed horizontally, and the top of the drop shaft was open to the atmosphere. *S*_*c*_ is the connected area between the dry and wet sides at the dividing wall bottom, and *h*_*w*_ is the test water depth. A horizontal pipe with a length of 0.3 m and a diameter of 0.05 m intersected the shaft vertically. As shown in Fig. 2, the horizontal pipe was connected to the wet side of the baffle-drop shaft. Furthermore, by rotating the whole dividing wall and baffles, the horizontal pipe could connect to the dry side. Three identical stainless-steel air chambers with a length of 0.5 m and a diameter of 0.05 m were connected in series with two valves, and the air chamber volume was *V*_*i*_ = 0.98 × 10^–3^ m^3^. For more reliable test results, ball valves were utilized in this experiment, which could be fully closed from fully opened by a quarter turn. A 0.3-m long metal hose was installed between the horizontal pipe and ball valve 1. The baffles at the shaft bottom bore a heavy water impact load during the geyser process. To study the force characteristics on the different baffles, three measuring points FT1, FT2, and FT3 (see Fig. 2) were selected, and their heights from the shaft bottom were 0.165, 0.245, and 0.325 m, respectively.

### Instrumentation

As shown in Fig. 2, six transducers were arranged in the shaft, where measuring point PT1 was used to test the water pressure of the horizontal pipe (*p*_*h*_), PT2 and PT3 were used to test the air pressure of the wet and dry sides at the shaft top (*p*_*w*_ and *p*_*d*_, respectively), and FT1, FT2, and FT3 were used to test the impact force on the different baffles (*F*_1_, *F*_2_, and *F*_3_, respectively). To achieve synchronous real-time acquisition, a SDA1000-SW-V02 (from Yufan Tech. Co., Ltd, Chengdu) data acquisition system was used in this test. The water and air pressures were measured using a high-frequency pressure transducer (MIK-P300, from Meacon Automation Tech. Co., Ltd, Hangzhou) with a maximum range of 10 kPa, an accuracy of ± 1.5% F.S, and a frequency of 50 Hz. The impact force on the baffles was measured using a force transducer (AT8106, from Autoda Automatic Equipment Co., Ltd, Suzhou) with a measuring range of 0–500 N, an accuracy of ± 0.2% F.S, and a frequency of 50 Hz. A digital air pump with a maximum range of 1.0 MPa and an accuracy of ± 0.005 MPa was used to inject the pressurized air into the air chamber. Pressure and force sensors were set to zero to reduce the error before each test. All sensors were calibrated by a professional company after 100 groups of tests to ensure measurement accuracy. Two Nikon® D850 high-speed video cameras (48 million pixels and 60 frames per second) were arranged in the test, one for recording the geyser process and the other located 10 m away from the drop shaft for estimating the geyser height (*h*_*g*_).

### Experimental procedure and conditions

It is very important to design an appropriate experimental procedure for simulating the geyser event, and the detailed procedure is described as follows:

1. Prepare the experiment before starting the geyser system, including mounting flanges and arranging transducers and cameras.
2. Close ball valve 1 and inject water into the shaft to the design water level. The opening or closing state of ball valves 2 and 3 is determined by the air volume of the test conditions.
3. Inject pressurized air into the air chamber to the design pressure. Close the air valve and keep the water quiescent.
4. Start the data acquisition from the video cameras and pressure transducers and record the digital air pump readings. Set all sensors to zero before each test.
5. Open Ball valve 1 manually as quickly as possible. According to multiple test statistics, control the average opening time of the valve to within 0.2 s.
6. After a sudden release of entrapped air for approximately 1–2 s, the geyser rises to the shaft top and sprays into the atmosphere.
7. After approximately 4–5 s, the geyser process is finished. Turn off the data acquisition system and video cameras after the pressure pulses disappear.
8. In preparation for the next test, open the flanges between the metal hose and horizontal pipe to drain the residual water in the shaft.

Four experimental variables were systematically varied to create different configurations in which high-pressure entrapped air would be released in baffle-drop shafts. The first variable was the initial water depth (*h*_*w*_) of the shaft, which was selected to assess whether a higher water level would favour geyser occurrence. The second variable was the initial air pressure (*p*_*i*_) of the air chamber, which was selected to assess whether stronger air pressurization would favour geyser occurrence. The third variable was the air volume (*V*_*i*_) of the air chamber, which was selected to assess whether a larger pressure gradient would favour geyser occurrence. The last experimental variable was the connected region area (*S*_*c*_) between the wet and dry sides, which was selected to assess the effect of area size on the impact load of the baffles.

Another potentially important variable was the connected mode of the horizontal pipe, which was switched from the wet side to the dry side by rotating the whole dividing wall and baffles. As presented in Table 1, a total of 202 different configurations were tested, and each group was repeated at least twice to ensure the consistency of the test results. Except for the four dimensionless variables shown in Table 1, the physical quantities analysed in the following are model values.

**Table 1:** Range of experimental variables considered in geyser experiments created by entrapped air release.

Variable	Range considered	Dimensionless range
Water depth	*h*_*w*_ = 0.20, 0.30, 0.40, and 0.50 m	*h*_w_* = *h*_*w*_/*H* = 0.167, 0.250, 0.333, and 0.417
Air pressure	*p*_*i*_ = 0.050, 0.100, 0.150, 0.200, 0.250, 0.300, 0.350, and 0.400 MPa	*p*_i_* = *p*_*i*_/*p*_*s*_ = 0.500, 1.000, 1.500, 2.000, 2.500, 3.000, 3.500, and 4.000
Air volume	*V*_*i*_ = 0.98, 1.96, and 2.94 × 10^–3^ m^3^	*V*_i_* = *nV*_*i*_/(*H*·*D*^2^) = 0.026, 0.052, and 0.078
Connected region area	*S*_*c*_ = 0, 0.005, 0.010, and 0.015 m^2^	*S*_c_* = 4*S*_*c*_/(π*D*^2^) = 0.000, 0.159, 0.318, and 0.478

Note: The standard atmospheric pressure (*p*_*s*_) is 101.325 kPa; the number of air chambers (*n*) is 1, 2, and 3.

## Experimental observations

Once the experimental runs were complete, the recorded movies were uploaded into a computer for visual analysis. By playing the video frame by frame and recording the vertical displacement of the air–water mixture surface within an elapsed time, the jetting velocity (*v*_*g*_) of the mixture and maximum geyser height (*h*_*g*_) were determined.

A general description of the geyser created by entrapped air release in baffle-drop shafts is given based on the experimental observations. After the ball valve opened, the high-pressure entrapped air mass was rapidly released in the air chamber with a loud noise. At this moment, the water remained transparent, but the water level in the shaft rose slightly. With the continuous release of the entrapped air mass, the water in the horizontal pipe was atomized and turned white. The free surface level in the vertical shaft started to rise rapidly. After the entrapped air mass was completely released, the water in the shaft was fully atomized, and the air–water mixture quickly moved under the inertia force. At this time, the mixing extent of air and water reached a maximum. Different from the dry side, due to the blockage of the baffles, the air–water mixture in the wet side could not continue to spray upwards when it moved to a nearly half height of the shaft. Immediately, the air–water mixture dropped step by step along the baffle edge, and fell back into the shaft bottom finally. However, the air–water mixture in the dry side continued to move upwards and rapidly sprayed into the atmosphere through the shaft top, forming a geyser. During this process, part of the water on the dry side spouted out of the shaft, while the other water fell back into the shaft and formed an inertial oscillation at the shaft bottom. At this moment, the mixing extent of air and water was dramatically decreased. A complete geyser process is provided in the supplementary video.

**Figure 3:**
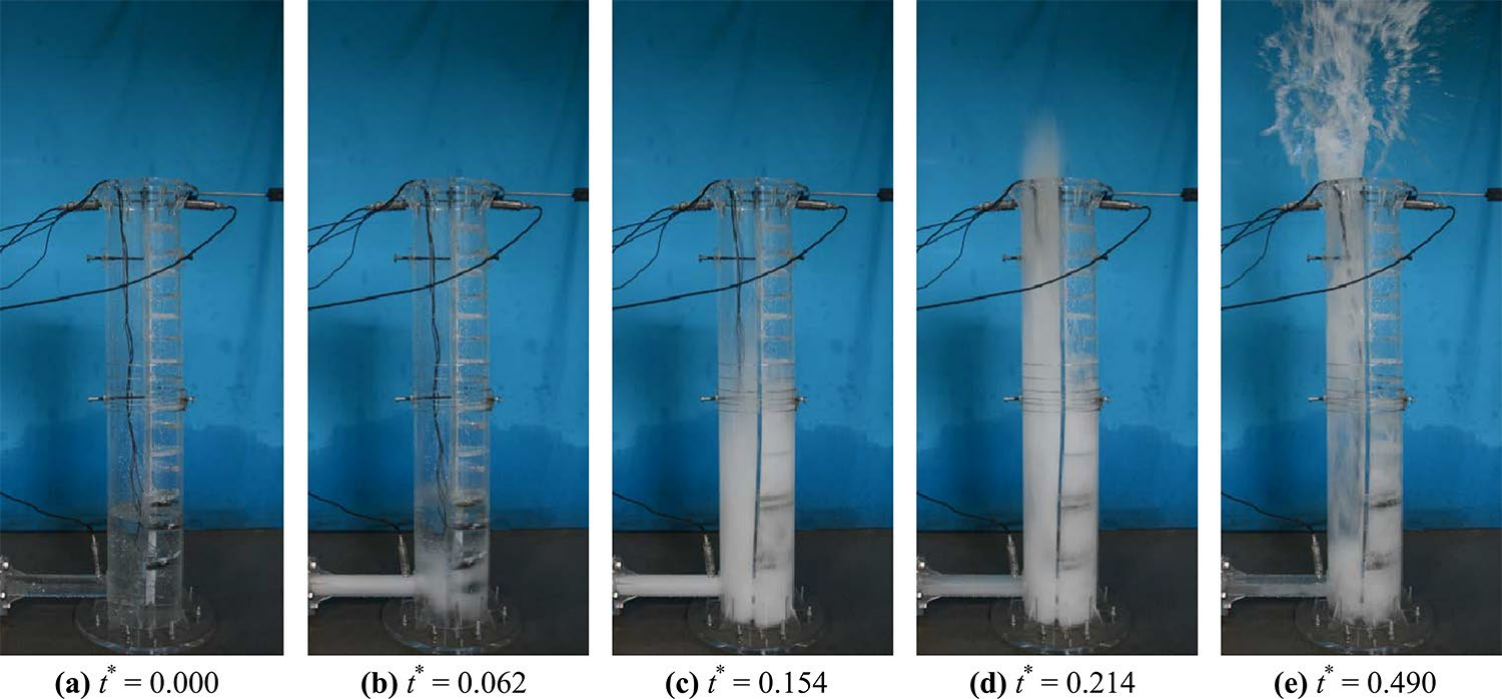
Typical flow regimes of the geyser during the entrapped air release.

The flow regimes of a geyser for a typical case (*h*_w_^*^ = 0.250, *p*_i_^*^ = 4.000, *V*_i_^*^ = 0.052, and *S*_c_^*^ = 0.478) are shown in Fig. 3, where dimensionless time t^*^ = *t*·*g*/*v*_gm_, *t* = geyser time, *g* = gravity constant, and* v*_gm_ = maximum jetting velocity of geyser. The valve was opened at t^*^ = 0.000, the high-pressure air mass was released, and the horizontal pipe was atomized at t^*^ = 0.062. When t^*^ = 0.154, the water in the shaft was fully atomized. Subsequently, the air–water mixture in the dry side sprayed to the shaft top at t^*^ = 0.214, and the geyser reached its maximum injection height when t^*^ = 0.490. The free surface of water in the shaft gradually calmed down when t^*^ = 2.242. By recording the vertical displacement of the air–water mixture surface, it was calculated that the maximum geyser velocity *v*_gm_ at the shaft top reached 10.65 m/s for the typical case shown in Fig. 3. Taking into account the air resistance effect, the vertical upwards parabolic motion was numerically computed by the following equation^26^:

1 $$h_{g} = 2.46\rho r\ln \left( {1 + \frac{{v_{0}^{2} }}{48.2\rho r}} \right)$$

where, *h*_*g*_ is maximum geyser height; *ρ* is air–water mixture density; *r* is air–water particles radius; *v*_0_ is initial velocity.

By calculation, the maximum geyser height reached 4.327 m at this jetting velocity. Moreover, the maximum jetting height estimated from the experimental video was 4.1 m, and this result closely approached the previous simulation value.

## Results and discussion

### Transient pressure

During the geyser process, significant changes in water and air pressure occurred in the baffle-drop shaft. The main reasons were the violent fluctuations in pressure caused by the release of high-pressure air masses and the local air pressure imbalances caused by the high-speed motion of the air–water mixture. Therefore, it is important to investigate the geyser phenomenon by analysing the pressure variation characteristics. In the test, it was found that the geyser occurrence had little effect on the air pressure of the wet side top (*p*_*w*_), which was only approximately 1/12–1/10 of the dry side pressure (*p*_*d*_) under the same test conditions. Thus, the pressure variation characteristics of the horizontal pipe and dry side are extensively analysed below.

**Figure 4:**
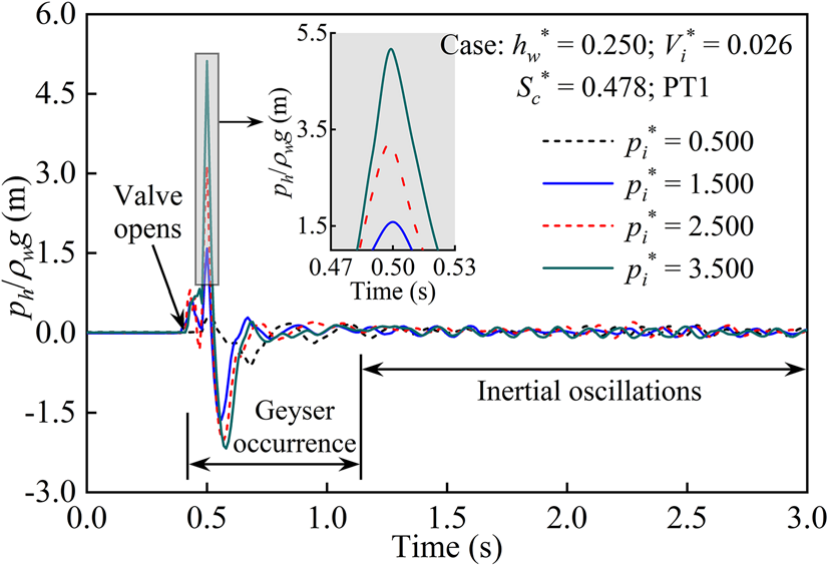
Sample pressure head of horizontal pipe from different air pressures.

Figure 4 shows the pressure variation law of the horizontal pipe for a set of typical cases (*h*_w_^*^ = 0.250; *V*_i_^*^ = 0.026; *S*_c_^*^ = 0.478), in which *p*_*h*_/*ρ*_*w*_*g* represents the pressure head. The initial hydrostatic pressure was subtracted, and the steady state could be clearly seen before the valve opens. The high-pressure entrapped air mass was released rapidly at *t* = 0.421 s, and the water was compressed in the horizontal pipe, resulting in a certain rise in the pressure head at PT1 that formed the first pressure peak. With the continuous entrapped air release, the flow velocity in the horizontal pipe increased, and the pressure decreased. Specifically, the first pressure trough appeared immediately after the first pressure peak. With the complete atomization of the water in the horizontal pipe, the high-pressure entrapped air was fully released, and the pressure head reached a maximum at *t* = 0.50 s. Immediately, the high-speed air–water mixture caused a significant reduction in pressure based on Bernoulli’s principle, and the pressure head dropped to a minimum at approximately *t* = 0.571 s. According to the video recording, the air–water mixture reached a maximum jetting height within approximately 0.567 s after the valve opened, and the total duration of the geyser process lasted approximately 0.718 s, corresponding to the time period of 0.421–1.139 s in Fig. 4. Subsequently, an inertial oscillation flow appeared for a long time, and the fluctuation range of the pressure head gradually decreased.

**Figure 5:**
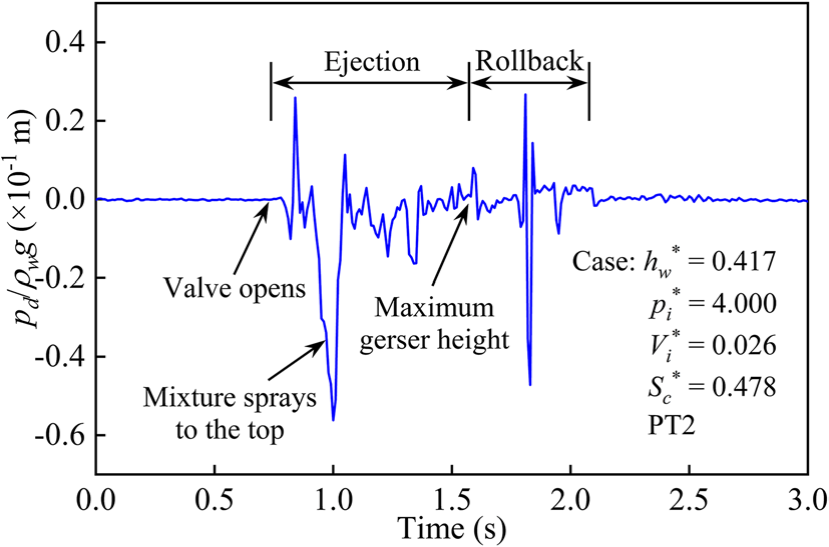
Sample pressure head of the dry side at shaft top.

Figure 5 presents a pressure head for the dry side measured at PT2 for a representative case (*h*_w_^*^ = 0.417; *p*_i_^*^ = 4.000; *V*_i_^*^ = 0.026; *S*_c_^*^ = 0.478). Obviously, a complete geyser process includes two stages: ejection and rollback. The high-pressure entrapped air mass rapidly released in the horizontal pipe after the valve opened at *t* = 0.738 s. The air–water mixture vertically jetted upwards with a high velocity, which caused the air in the shaft to be compressed and the pressure head of the dry side to increase. With continuous motion, the free surface of the air–water mixture reached the shaft top at *t* = 0.971 s. Then, the air pressure of the shaft continuously decreased, and the pressure head dropped to a minimum at *t* = 1.001 s. Affected by gravity and air resistance, the vertical upwards motion of the air–water mixture reached the maximum geyser height at *t* = 1.573 s, at which time the pressure head measured at PT2 dropped to zero. For the air–water mixture jetted into the atmosphere, part of it sprayed out of the shaft, and the rest fell into the shaft. During the rollback process, the air on the dry side was compressed by the falling air–water mixture, and the other pressure peak appeared at *t* = 1.811 s. Because the air was dragged down by the falling water, a negative pressure was immediately formed at PT2. Subsequently, all the air–water mixture fell back to the shaft bottom at *t* = 2.079 s in Fig. 5, and the geyser process ended.

Based on the time nodes of each stage during the geyser process in Fig. 5, for this typical case, the duration of ejection and rollback stages were 0.835 s and 0.506 s, respectively, and the total duration of the geyser process lasted 1.341 s. Comparing the two geyser processes shown in Figs. 4 and 5, the total duration of the former case is 0.718 s, which is significantly shorter than the latter case. It can be seen that the water depth (*h*_w_^*^ = 0.417) and air pressure (*p*_i_^*^ = 4.000) of the case in Fig. 5 are both greater than those in Fig. 4. Therefore, for the case in Fig. 5, more air–water mixture formed in the shaft and the higher jetting height in the atmosphere, which resulted in a longer geyser duration. These variation laws also showed that several variables, including water depth and air pressure, synergistically determined the duration of a geyser process.

### Geyser height

The geyser process and pressure variation characteristics of the baffle-drop shaft are analysed above, but the harmful hydraulic phenomenon of the geyser has not been precisely defined. After reviewing the current related research, there is no exact definition of a geyser. To facilitate the comprehensive investigation of the formation conditions of geysers, a clear definition of a geyser was proposed in this paper based on geyser event recordings, experimental observations, and geyser hazards. That is, a violent phenomenon can be described as an air–water mixture being sprayed into the atmosphere from the shaft due to the release of a high-pressure entrapped air mass in the main tunnel. However, even if an entrapped air mass is released in a deep tunnel drainage system, there may be no water sprayed into the atmosphere and no effect on ground safety. Therefore, this phenomenon is not a geyser.

**Figure 6:**
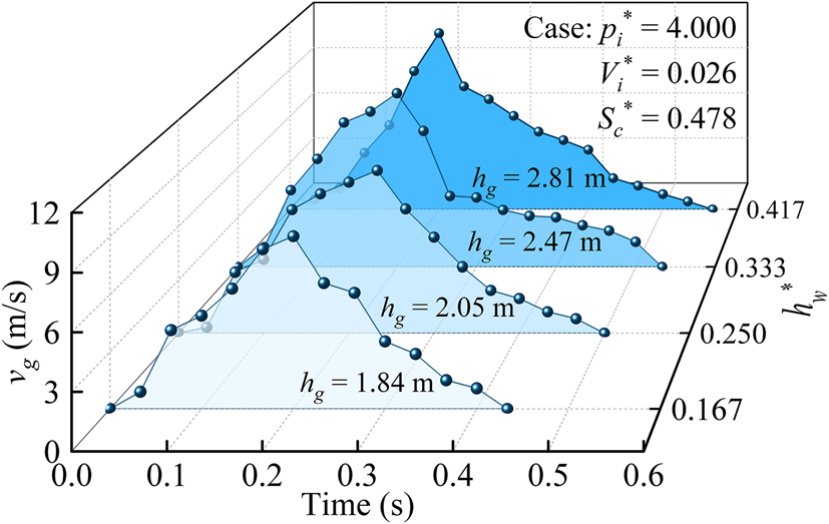
Jetting velocity time history curves of the air–water mixture.

Combined with the above definition of a geyser, the jetting velocity (*v*_*g*_) of the air–water mixture and maximum geyser height (*h*_*g*_) were obtained by visual analysis of the experimental videos. Fig. 6 presents a set of jetting velocity time history curves of the air–water mixture under different water depths. Obviously, the jetting velocity of the air–water mixture rapidly increased after the entrapped air release. The free surface reached the shaft top at *t* = 0.15–0.20 s and spouted out of the shaft with a velocity of 9.023–11.347 m/s. Subsequently, the jetting velocity began to gradually decrease due to the air resistance and gravity. Until the velocity dropped to zero, the geyser height of the air–water mixture reached the maximum, and the shaded area illustrated in Fig. 6 was the maximum geyser height (*h*_*g*_).

In addition, by comparing the geyser process under different water depth conditions (0.167 ≤ *h*_w_^*^ ≤ 0.417), it was found that for a constant air pressure (*p*_i_^*^ = 4.000), the maximum jetting velocity and maximum geyser height of the air–water mixture both increased with increasing water depth. The likely reason for this variation is that the air concentration of the air–water mixture decreases with increasing water content. For the same air pressure condition, the greater the water proportion is, the smaller the air resistance in the atmosphere, and the higher the jetting height during the geyser process.

Through the synthetical analysis of the experimental phenomena and monitoring data, the results show that the water depth, air pressure, and air volume synergistically determined the geyser intensity. Table 2 lists the maximum jetting velocity and maximum height of the geyser under different conditions, in which *h*_*g*_ is the vertical height from the bottom of the shaft. Among them, the geyser phenomenon occurred in serial numbers 1 to 18, and the geyser intensity was positively correlated with the water depth, air pressure, and air volume. Experimental variables of serial numbers 19–24 are the critical conditions; that is, a geyser can be caused by increasing any variable of the water depth, air pressure, and air volume. In addition, the Reynolds number R = *v*_*gm*_*h*_*g*_/*υ* and Weber number W = (*ρv*_*gm*_^2^*h*_*g*_/*σ*) of Serial number 1–18 are listed in Table 2, where *υ* = water kinematic viscosity, *ρ* = water density, and *σ* = water surface tension. As stated in Table 2, the calculated two numbers limit around R = 17–54 × 10^5^ and W^0.5^ = 473–940. These results satisfy the relevant limitations of using Froude similitude for high-speed air–water two-phase flows, where R > 2–3 × 10^5^ or W^0.5^ > 140^27^.

**Table 2:** Maximum jetting velocity and geyser height under different conditions.

Serial number	*h*_w_*	*p*_i_*	*V*_i_*	*v*_*gm*_ (m/s)	*h*_*g*_ (m)	R (× 10^5^)	W^0.5^	Serial number	*h*_w_*	*p*_i_*	*V*_i_*	*v*_*gm*_ (m/s)	*h*_*g*_ (m)	R (× 10^5^)	W^0.5^
1	0.167	4.000	0.026	9.023	1.84	17.0	472.9	13	0.250	2.000	0.052	9.886	2.23	28.0	634.6
2	0.250	3.500	0.026	8.534	1.65	24.2	547.8	14	0.250	2.500	0.052	10.807	2.67	30.6	693.7
3	0.250	4.000	0.026	9.146	2.05	25.9	587.1	15	0.250	3.000	0.052	11.574	3.03	32.8	743.0
4	0.333	3.000	0.026	8.703	1.74	32.8	645.1	16	0.250	1.000	0.078	8.369	1.56	23.7	537.2
5	0.333	3.500	0.026	9.839	2.20	37.1	729.3	17	0.250	1.500	0.078	10.117	2.34	28.6	649.5
6	0.333	4.000	0.026	10.487	2.47	39.6	777.3	18	0.250	2.000	0.078	10.958	2.69	31.0	703.4
7	0.417	2.000	0.026	7.535	1.33	35.5	624.5	19	0.167	3.500	0.026	Critical condition			
8	0.417	2.500	0.026	8.245	1.54	38.9	683.3	20	0.250	3.000	0.026				
9	0.417	3.000	0.026	8.827	1.79	41.6	731.5	21	0.333	2.500	0.026				
10	0.417	3.500	0.026	10.303	2.42	48.6	853.9	22	0.417	1.500	0.026				
11	0.417	4.000	0.026	11.347	2.81	53.5	940.4	23	0.250	1.000	0.052				
12	0.250	1.500	0.052	8.355	1.57	23.6	536.3	24	0.250	0.500	0.078				

Based on the above research conclusions, the geyser intensity was synergistically determined by three variables: water depth (*h*_w_^*^), air pressure (*p*_i_^*^), and air volume (*V*_i_^*^). Therefore, the maximum geyser height *h*_*g*_ can be written as:

2 $$h_{g} = f\left( {h_{w}^{*} ,p_{i}^{*} ,V_{i}^{*} } \right)$$

Equation (2) can be rewritten not dimensionally as follows:

3 $$\frac{{h_{g} }}{D} = k_{1} \left( {h_{w}^{*} } \right)^{{k_{2} }} \left( {p_{i}^{*} } \right)^{{k_{3} }} \left( {V_{i}^{*} } \right)^{{k_{4} }}$$

where *k*_1_, *k*_2_, *k*_3_, and *k*_4_ are unknown parameters.

Combined with the test results listed in Table 2, a mathematical model among *h*_*g*_/*D*, *h*_w_^*^, *p*_i_^*^, and *V*_i_^*^ was established by using the multiple linear regression model, and the fitting formula of the maximum geyser height was then obtained:

4 $$\frac{{h_{g} }}{D} = 158.021\left( {h_{w}^{*} } \right)^{0.421} \left( {p_{i}^{*} } \right)^{0.991} \left( {V_{i}^{*} } \right)^{0.959}$$

in which *D* is the baffle-drop shaft diameter.

**Figure 7:**
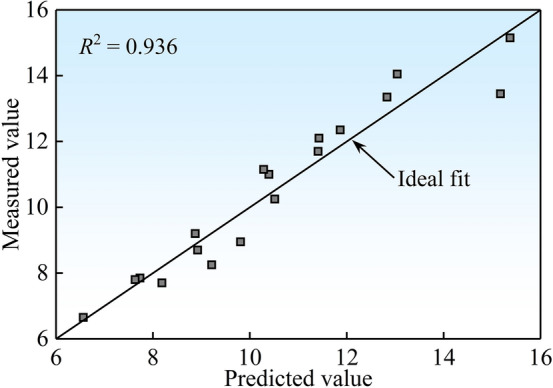
Comparison of predicted and measured values of *h*_*g*_/*D*.

It is cautioned that Eq. (4) is limited to the experimental conditions of the present study, which are 0.167 ≤ *h*_w_^*^ ≤ 0.417, 1.000 ≤ *p*_i_^*^ ≤ 4.000, and 0.026 ≤ *V*_i_^*^ ≤ 0.078. To verify the accuracy of Eq. (4), Fig. 7 illustrates a comparison result between the predicted and measured values of the dimensionless maximum geyser height. The goodness of fit in Eq. (4) has an *R*^2^ value of 0.936, which indicates a good fit to the data.

**Figure 8:**
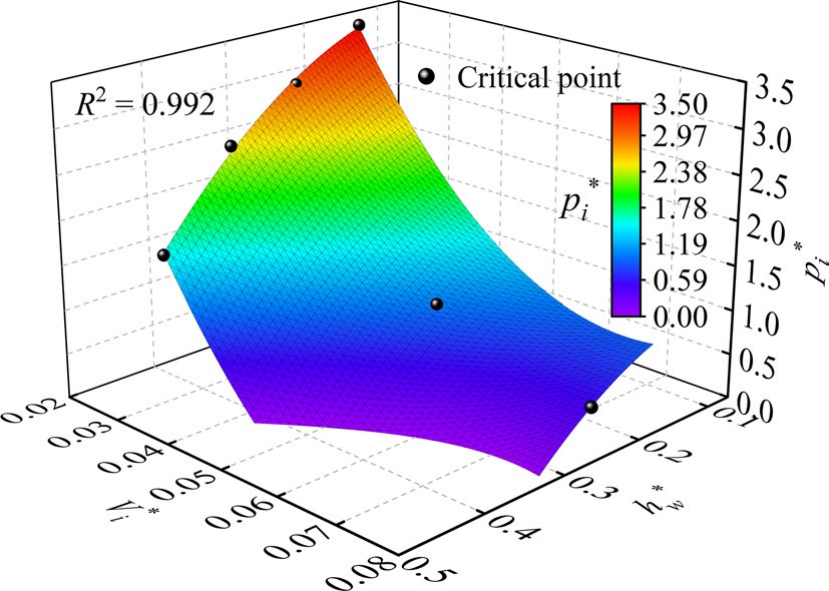
Critical conditions for a geyser.

In addition, based on the critical conditions of the geyser listed in Table 2, a parabolic 2D model was adopted to nonlinearly fit the surface equation of discrete points, and a 3D surface graph is shown in Fig. 8. Combined with the response relationship between the ejection intensity and water depth, air pressure, and air volume during a geyser event, a governing equation of a geyser for a baffle-drop shaft is proposed as follows:

5 $$p_{i}^{*} > 1163.769V_{i}^{*2} - 17.733h_{w}^{*2} - 170.521V_{i}^{*} + 2.551h_{w}^{*} + 7.191$$

Equation (5) is also limited to the test conditions of this study. If the relationships among *h*_w_^*^, *p*_i_^*^, and *V*_i_^*^ satisfy Eq. (4), a geyser phenomenon will occur. To verify the reliability of Eq. (5), various experimental variables, including geyser and no geyser, were substituted into the equation. The results show that a geyser occurred for the experimental variables satisfying Eq. (5), and vice versa. Therefore, Eq. (5) can be used as a requisite condition to judge whether a geyser occurs.

### Impact load on baffles

After the high-pressure entrapped air mass was released, a great deal of the air–water mixture rushed into the wet side of the shaft and caused a considerable impact load on the bottom baffles. This could be catastrophic if the baffles were damaged and the deep tunnel system could not operate normally. To fully investigate the load level and distribution during a geyser event, three baffles (measuring points FT1, FT2, and FT3 in Fig. 2) were selected as research objects to analyse the influence of air pressure, air volume, and water depth on the baffle load characteristics. The impact load values in the following were measured under the condition of a horizontal pipe connecting to the wet side.

**Figure 9:**
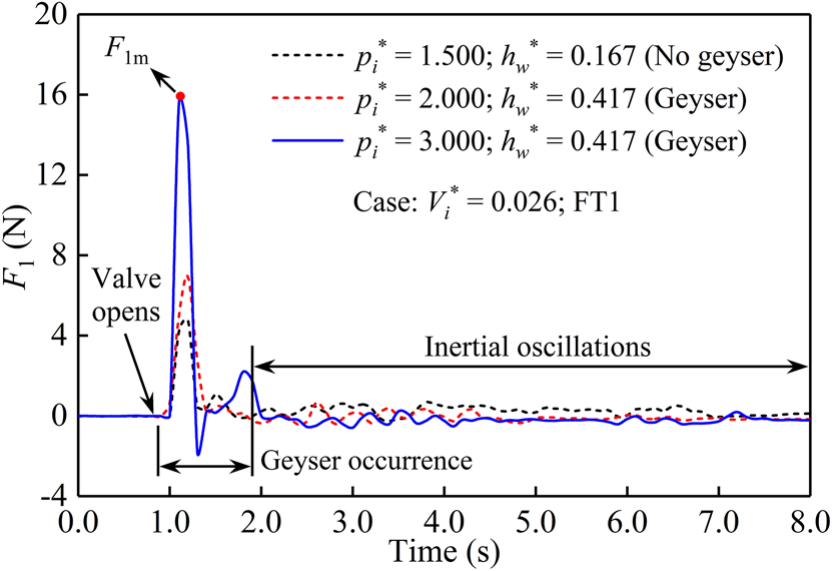
Time history curves of the water impact force on the baffle.

Figure 9 presents load results measured at FT1 when the air mass release occurred for three representative cases, in which a geyser occurs in two cases and no geyser occurs in the other case. Obviously, the impact force on the baffle increased sharply during the geyser occurrence and rapidly reached its maximum value *F*_1m_. Immediately after entering the inertial oscillations stage, the bottom baffles vibrated with the oscillatory flow. Comparing these three cases, the maximum impact load on the baffle during the geyser event is significantly greater than that without a geyser. To facilitate the guidance of engineering practice, the maximum impact forces measured on FT1, FT2, and FT3 were converted into the average surface pressures *P*_1m_, *P*_2m_, and *P*_3m_, respectively, by dividing by a baffle area.

**Figure 10:**
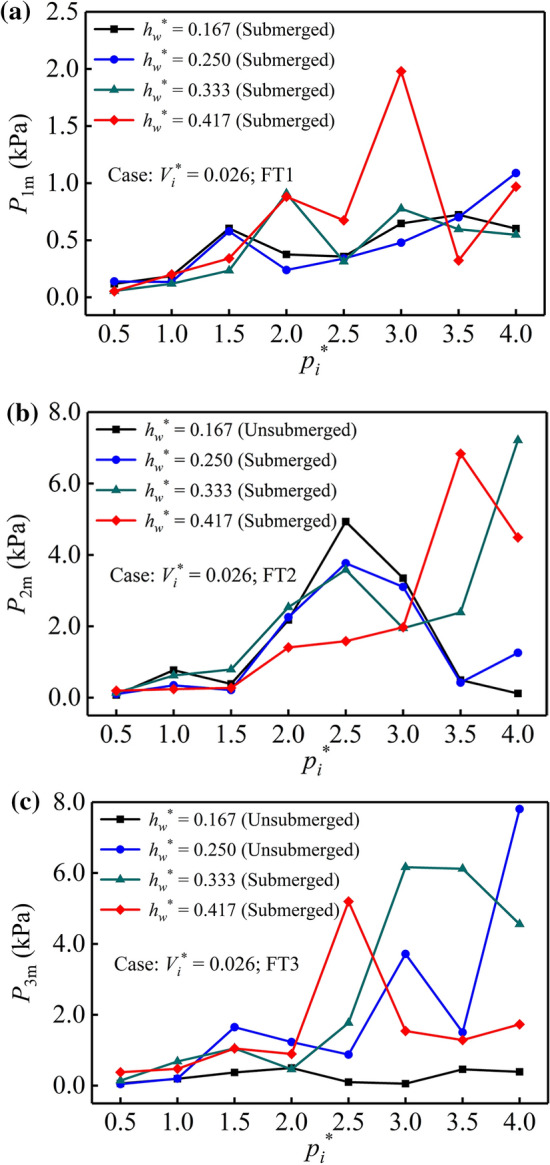
Maximum water impact pressure on the measuring baffles under different pressures.

The variation law of the maximum impact load on the baffles at FT1, FT2, and FT3 under different pressures is illustrated in Fig. 10. It can be seen in the comparison that the trend of impact pressure on the baffles generally increases with increasing air pressure, but there are still individual negative correlations. The main reason for this variation is that the rapid process of the high-speed air–water mixture impacting the baffles is random. As the other conditions are certain, if the amount of air–water mixture impacting the baffle is large, the impact load borne by the baffle is also larger. In contrast, the smaller the air–water mixture is, the smaller the impact force on the baffle. Another reason may be that the impact load is related to whether the baffle is submerged. If the baffle was below the water surface during the geyser, the rising air–water mixture bore the upper water pressure, which weakened the impact force on the baffles. However, for the unsubmerged baffles, a certain vertical height provides an acceleration space for the air–water mixture. The baffles would experience a considerable impact force when the critical surface between the air and air–water mixture jetted to the baffle bottom. Combining the above two reasons, it is apparent that the maximum impact load on the baffles presents a significant fluctuation trend under different air pressures.

Comparing the maximum impact load on FT2 and FT3 under the same air pressure (*p*_i_^*^ = 2.500) and submerged conditions in Fig. 10b and c, it is obvious that the pressure values of FT2 and FT3 are *P*_2m_ = 3.584 kPa and *P*_3m_ = 1.771 kPa at *h*_w_^*^ = 0.333. This shows that the amount of air–water mixture forcing on FT2 is significantly greater than that on FT3. However, when *h*_w_^*^ = 0.417, the pressure values of FT2 and FT3 are *P*_2m_ = 1.583 kPa and *P*_3m_ = 5.197 kPa. For this case, the amount of air–water mixture forcing on FT3 is significantly greater than that on FT2, and the variation is the opposite of the former. The above two situations further showed that the process of the air–water mixture impacting the baffle bottom presents a high randomness during the entrapped air release.

For the same air pressure (*p*_i_^*^ = 2.500) and different submerged conditions at FT2 in Fig. 10b, the baffle is not submerged when *h*_w_^*^ = 0.167, and the maximum impact load is *P*_2m_ = 4.933 kPa. When *h*_w_^*^ = 0.250, 0.333, and 0.417, the pressure values of the submerged FT2 are 3.766, 3.584, and 1.583 kPa, respectively. The maximum impact loads of these three cases are all smaller than that of the unsubmerged baffle. In addition, comparing the maximum impact load on FT3 under the same air pressure (*p*_i_^*^ = 4.000) and different submerged conditions in Fig. 10c, it can be seen that the pressure value of the unsubmerged FT3 can reach 7.908 kPa. However, when *h*_w_^*^ = 0.333 and 0.417, measuring point FT3 is submerged, and the pressure values are 4.555 kPa and 1.730 kPa, respectively, which are significantly less than the load of the former unsubmerged baffle. These two cases illustrate that whether the baffle is submerged or not has a great influence on the impact load of the baffle bottom.

Thus, it can be seen that the impact load on the baffle bottom during the geyser process is not only related to the air pressure, measuring point position, and baffle submerged state but also closely related to the randomness of the air–water mixture jetting on the baffle. Therefore, it is difficult to predict the water impact load on the baffles under different conditions through a mathematical model. However, previous research results have shown that the maximum water impact load of the upper inflow on the baffles is approximately 10.88–34.02 kPa during a normal discharge process^28^. In the case of a geyser occurrence, the maximum impact load borne by the baffle bottom can reach 99.01–395.38 kPa (converted into prototype values), which is approximately 10 times the hydrodynamic load under normal discharge conditions. Therefore, it is necessary to improve the strength and stiffness of the baffles at the shaft bottom during structural design to avoid baffle structural failure during geyser events.

## Conclusions and future work

During geyser events, drop shafts and baffle structures bear strong impact loads generated by the release of high-pressure entrapped air masses, which might even cause failures in deep tunnel drainage systems. In this paper, an atmospheric scale model test was carried out to investigate the response relationship between a geyser mechanism and its influencing factors, such as water depth, inlet pressure, and inlet volume, and the main conclusions of this study are summarized as follows.

1. There are two reasons for the violent fluctuation of water and air pressure in a baffle-drop shaft during a geyser event. One is caused by the release of a high-pressure entrapped air mass, and the other is the local air pressure imbalance caused by the high-speed motion of the air–water mixture. Under a certain water depth condition, the air pressure and volume have a greater impact on the pressure in the shaft. However, for a shallow water depth, the high-pressure entrapped air mass can be released due to an insufficient air–water mixture, and a geyser is not formed.
2. According to geyser event recordings, experimental observations, and hazards, a geyser phenomenon for a baffle-drop shaft was redefined, and a prediction formula of the maximum geyser height was established by adopting a multiple linear regression model. In addition, combined with the response relationship between the ejection intensity and water depth, air pressure, and air volume during a geyser event, a governing equation of a geyser for a baffle-drop shaft was proposed. Through comparison and verification, it was found that these two models could satisfactorily predict geyser height and accurately judge whether a geyser occurs.
3. Except for the air pressure, measuring point position, and baffle submerged state, the impact load on the baffles at the shaft bottom was also related to the randomness of the air–water mixture jetting on the baffle during a geyser event. The experimental data showed that the maximum impact load borne by a baffle bottom in a geyser occurrence was approximately 10 times the hydrodynamic load on a baffle under normal discharge conditions. To avoid baffle structural failure, it is essential to improve the strength and stiffness of the baffle at the shaft bottom.
4. Although a series of model tests were carried out to study the geyser phenomenon, pressure characteristics, and baffle impact loads of a baffle-drop shaft and some valuable results were obtained, some limitations still exist. The geyser model test in this paper was carried out under standard atmospheric pressure conditions. Due to the scale effect, the experimental air pressure cannot be reduced equivalently, and some limitations in quantifying and evaluating the flow characteristics of water entrainment aerodynamics existed in this physical test. Therefore, it is necessary to carry out prototype numerical simulations and subatmospheric tests on the geyser characteristics of baffle-drop shafts. In addition, considering the impact of the normal discharge process on the baffle hydrodynamic load, the geyser model test of the baffle-drop shaft during the discharge process will be carried out in the next step.

## Supplementary Information

Supplementary Video 1.

## Data Availability

The datasets used and/or analysed during the current study are available from the corresponding author on reasonable request.
